# Understanding the genetics and neurology: an overview of adult neurogenetics

**DOI:** 10.2478/abm-2025-0022

**Published:** 2025-09-02

**Authors:** Pasin Hemachudha, Prakit Anukoolwittaya, Thanakit Pongpitakmetha, Yutthana Joyjinda, Chanida Ruchisrisarod, Abhinbhen W. Saraya, Wanakorn Rattanawong, Poosanu Thanapornsungsuth, Thiravat Hemachudha

**Affiliations:** Thai Red Cross Emerging Infectious Diseases Health Science Centre, King Chulalongkorn Memorial Hospital-The Thai Red Cross Society, Bangkok 10330, Thailand; Division of Neurology, Department of Medicine, Chulalongkorn University, King Chulalongkorn Memorial Hospital, Faculty of Medicine, Chulalongkorn University, Bangkok 10330, Thailand; Comprehensive Headache and Orofacial Pain (CHOP) Research Group, Faculty of Medicine, Chulalongkorn University, Bangkok 10330, Thailand; Department of Pharmacology, Faculty of Medicine, Chulalongkorn University, Bangkok 10330, Thailand; Chula Neuroscience Center, King Chulalongkorn Memorial Hospital, The Thai Red Cross Society, Bangkok 10330, Thailand; Department of Medicine, King Mongkut's Institute of Technology Ladkrabang, Bangkok, Thailand; Center of Excellence in Integrative Medicine and Public Health, College of Eastern Medicine, Rangsit University, Pathum Thani 12000, Thailand

**Keywords:** adult, genetic, gene therapy, mitochondrial disorders, neurogenetics, neurology, next generation sequencing

## Abstract

Neurogenetics investigates the genetic basis of neurological disorders. It encompasses conditions ranging from neurodegenerative diseases with predominantly polygenic risk genes, such as Alzheimer's and Parkinson's, to monogenic diseases and repeated expansion disorders within movement and neuromuscular disorders, such as Friedreich ataxia and muscular dystrophies. Significant advances in recent years that have revolutionized our understanding of disease mechanisms and paved the way for personalized medicine approaches are due to the field of neurogenetics, with its intricate relationship both with clinical and genetic research. Therefore, all neurologists, even in resource-limited settings, are aware of the critical genetic basis; standard molecular diagnostic techniques such as next-generation sequencing, whole exome, and whole genome sequencing; and possible therapeutic modalities of their field. This review will also touch on elements of the neurogenetic clinic in tertiary care, ethical considerations, and insight into ongoing research that would help improve patient care and enhance clinical outcomes.

Neurogenetics represents two scientific disciplines: neurology, that is, the study of the nervous system and its disorders; and genetics, that is, the exploration of the hereditary that defines an individual. Neurogenetics studies the genetic underpinnings of various neurological conditions, from monogenic disease, which is relatively easy to understand, to polygenic diseases with vast genetic heterogeneity.

The impact of neurogenetics on the practice of neurology has led to an understanding of the etiology and pathophysiology of neurological disorders and has also revolutionized diagnostic approaches, prognostic assessments, and therapeutic strategies. With advanced genomic technologies such as next-generation sequencing (NGS), identifying disease-causing genetic mutations has become more precise and accessible. This, in turn, has translated into earlier diagnoses, improved genetic counseling, and the development of targeted therapies.

This review aims to provide neurologists with a genetic basis for neurological disorders, diagnostic advancements, current and future therapeutic interventions, and research. It will also examine how neurogenetics empowers neurologists to provide personalized medicine and treatments to individual genetic profiles, shaping the future of neurological care.

## Overview of human DNA

Deoxyribonucleic acid (DNA) is the genetic blueprint for all living organisms. It carries the instructions necessary for an individual's growth, development, and functioning and also stores and passes on genetic information to their offspring. The human DNA consists of two long polynucleotide strands of repeating units called nucleotides that coil around each other to form a double-helix structure resembling a twisted ladder. Each nucleotide consists of the DNA strand's sugar (deoxyri-bose) backbone with a phosphate group attached to each sugar, forming a phosphate-sugar backbone. There are four types of nitrogenous bases in DNA: adenine (A), thymine (T), cytosine (C), and guanine (G). These bases pair together in a complementary fashion, with adenine always pairing with thymine and cytosine always pairing with guanine via hydrogen bonds.

The DNA is organized into segments called genes, which contain specific sequences of nucleotides that encode instructions for synthesizing proteins or functional RNA molecules. Each gene is the fundamental unit of heredity that determines an individual's characteristics, and its transcription and translation allow the flow of genetic information from the DNA to the functional proteins. During transcription, the DNA template is utilized by RNA polymerase to synthesize a complementary RNA molecule, forming a pre-messenger RNA (pre-mRNA) transcript containing both introns and exons, which carry the genetic code from the DNA. The pre-mRNA undergoes processing steps, including splicing, to remove the intronic region, a noncoding region by the spliceosome. The spliceosome recognizes conserved nucleotide sequences at the exon–intron boundaries, allowing for precise excision of introns and subsequent joining of exons, which is the coding region. Alternative splicing further enhances the complexity of gene expression by generating multiple mRNA isoforms from a single gene, thereby influencing protein diversity and function.

The mature mRNA derived from exons is then transported to the cytoplasm, where translation occurs. Translation involves decoding the mRNA sequence by ribosomes, with transfer RNA (tRNA) molecules bringing specific amino acids to the ribosome based on the mRNA codons. These amino acids are linked in a particular order to form a polypeptide chain, ultimately folding into a functional protein.

The complete set of genetic material, including all its genes, is the genome and consists of approximately 3 billion DNA base pairs [1]; 99% of the human genome consists of introns, so they do not contribute directly to protein synthesis. Yet, they are essential in gene regulation and control of gene expression and chromosomal structure, and they regulate cellular processes [2]. The human DNA is organized into 46 chromosomes, 1 chromosome from each pair inherited from each parent. The first 22 pairs are autosomes, while the 23rd determines an individual's sex.

**Figure 1.**
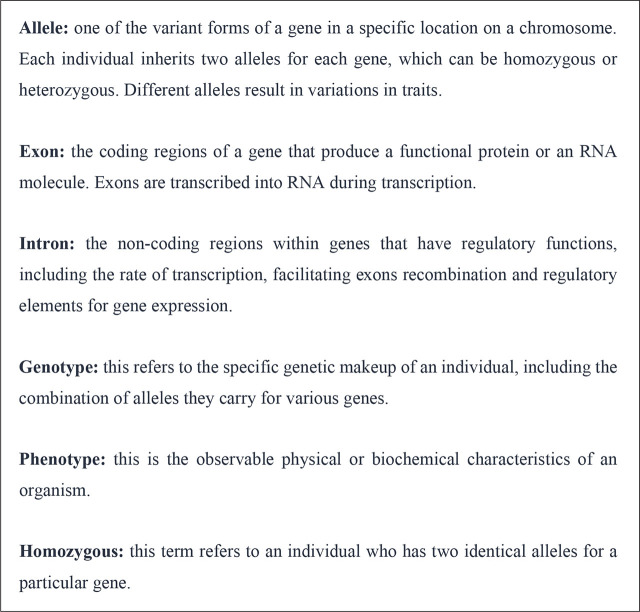
Commonly encountered genetic terms.

In summary, the DNA becomes genes, a unit that encodes instructions for protein synthesis. Genes are arranged into chromosomes, and 46 chromosomes become the genome. The standard genetic terms are summarized in **Figure 1**.

## Genetic disorders in neurology: from genes to clinical manifestations

Genetic disorders in neurology encompass a diverse pathology resulting from mutations in various genes. These disorders often present with complex clinical phenotypes, and both monogenic and polygenic diseases affect individuals across the lifespan. Neurologists with a knowledge of genetic disorders and genes can understand the mechanisms and clinical manifestations and reach a definitive diagnosis.

Most diagnoses are usually made through evaluation of the history and progression of the disease. Physical examination must be done in the context of the history, while serial examinations based on the symptoms may initially be equivocal. Finally, laboratory and special tests such as cerebrospinal fluid analysis, neuroimmunology study, imaging study, elect-rodiagnosis, or electroencephalography confirm the diagnosis.

However, things can become complicated when the investigation turns out to be negative. Further probe into three generations of the family history, including a condition that occurs in two or more relatives at a relatively younger onset, including ethnic background and consanguinity, may suggest a genetic predisposition. Additionally, it becomes necessary to examine for a combination of clinical features or dysmorphic features, which may provide clues for hereditary disease. Even with a negative family history in adults, genetic conditions should not be ruled out as *de novo* mutation can occur. Evaluation by a geneticist may be helpful, especially before the advent of advanced genetic sequencing, and the genes have to be carefully selected for examination. Extensive investigations can sometimes include repeated lumbar punctures and repeated imaging, and the time to diagnosis of genetic diseases can even take several years; thus, the patient and relatives might suffer from living life with an unknown disease condition.

**Figure 2.**
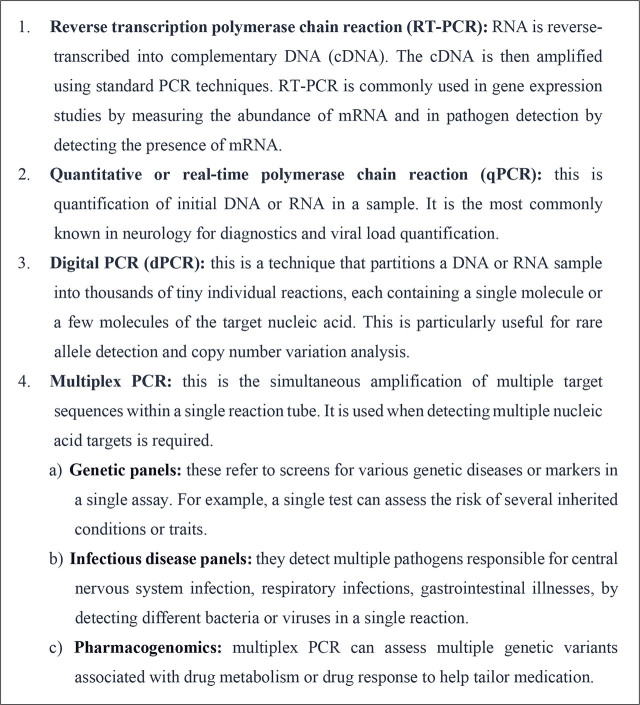
Commonly encountered NAATs. cDNA, complementary DNA; dPCR, digital PCR; NAAT, nucleic acid amplification tests; qPCR, quantitative or real-time polymerase chain reaction; RT-PCR, reverse transcription polymerase chain reaction.

Nucleic acid amplification tests (NAAT) are a group of molecular techniques for detecting and quantifying nucleic acids, such as DNA or RNA, and are widely used in medicine, including in diagnostics and research. Polymerase chain reaction (PCR) is a branch of NAATs and involves amplifying a specific DNA segment using a polymerase enzyme, thermal cycling, and specific DNA primers. PCR can be used for DNA quantification, genetic analysis, and pathogen detection [3]. The standard sequencing techniques are summarized in **Figure 2**.

Mutations in the DNA span from single base pair alterations and repeat expansions to deletions or duplications of exons or entire genes, with the latter resulting in substantial genetic modifications often associated with deleterious effects. Childhood neurogenetics represents a significant domain within neurogenetics disorders, encompassing a spectrum of conditions. These include neurodevelopmental disorders characterized by congenital malformations such as agenesis or polymicrogyria, developmental delay, and cognitive impairment; genetic epilepsy contributing to developmental and epileptic encephalopathy alongside diverse seizure types, as well as recognized epilepsy syndromes such as Dravet and Lennox-Gastaut syndrome; neuromuscular disorders including congenital myopathies, muscular dystrophies, and numerous hereditary neuropathies; neurocutaneous syndromes such as neurofibromatosis, tuberous sclerosis, and Sturge–Weber Syndrome; as well as mitochondrial and neurometabolic disorders. Managing patients with these conditions typically entails a multifaceted approach that is heavily reliant on collaboration between healthcare professionals and the patient's caregivers. Several well-documented childhood neurogenetic disorders may manifest at any point in life, including adolescence and adulthood; however, these fall beyond the scope of this review and have been extensively addressed elsewhere [4].

In adult neurogenetics, mutations often affect a single gene in the coding region. These changes can be due to changes in single base pairs or insertions/deletions of one or multiple base pairs. Single base pair substitutions, called point mutations, result when another replaces a single base pair. Deletions or insertions of base pairs represent another mutation. Deletions/insertions that are not a multiple of three will alter the reading frame and the resulting amino acid sequence downstream, recognizing a premature stop codon and shortened polypeptides. Despite changes, most amino acid changes are not pathogenic, as substituting similar amino acids will allow functional proteins.

The advent of advanced genetic testing platforms is a clear advantage over Sanger sequencing. The physician can select multiple genes associated with certain presentations (syndromic panel), which can diagnose 30%–70% of undiagnosed cases [5, 6]. Moreover, whole exome sequencing can provide a further definitive diagnosis in 20%–45% of cases, but may require input from neurogeneticists as most amino acid changes are not pathogenic and may be of uncertain significance. The variant of uncertain significance (VUS) is the genetic variation that is uncertain or unclear about whether that variant is connected to a certain condition; this has to be interpreted carefully by the trained geneticist. Publicly available databases on pathogenic mutation have led to a much more accurate analysis of mutations [7]. Using the clinical syndrome to select a comprehensive panel, a general neurologist can arrange for the patient to be tested without needing geneticist input. The article will illustrate the wide spectrum of neurological diseases from neurode-generative, movement disorders, neurovascular diseases, and neuromuscular diseases to epilepsy, which are associated with genetic mutations across different age groups from adolescence onwards, which may largely depend on the gene affected.

### Alzheimer's disease

The most well-known neurodegenerative disease with pathogenesis is centered on excessive amyloid-beta precursor protein gene (*APP*) on chromosome 21 and the beta-amyloid cascade hypothesis. The disease commonly manifests with progressive episodic and later semantic memory loss. Procedural memory and motor skills are usually preserved until very late. Alzheimer's disease (AD) is influenced by multiple genes, with early onset, usually from monogenic change, and either monogenic or polygenic changes influence late onset presentation. However, ongoing research has discovered several additional risk genes and genetic variants that do alter the disease trajectory.

### Early-onset Alzheimer's disease

Early-onset Alzheimer's disease (EOAD) occurs in individuals under the age of 65 and accounts for 6% of all AD, whereas monogenic AD accounts for only 1%. The importance of genetic factors is not clearly understood in the remaining EOAD [8]. Mutations in three specific genes, *APP*, presenilin-1 (*PSEN1*), and presenilin-2 (*PSEN2*), are strongly associated with EOAD. These genes are autosomal dominant with almost complete penetrance.

### LOAD

Late-onset Alzheimer's disease (LOAD) is the more common form of Alzheimer's, occurring in individuals aged 65 and older. Several genetic risk factors have been identified, which are more complex than EOAD. The most well-known genetic risk factor for LOAD is the apolipoprotein E (*APOE*) gene. There are three common alleles of the APOE gene: E2, E3, and E4. The E3 allele is the wild type or normal variant. The E4 allele is associated with an increased risk of AD; carriers of one allele are twofold to threefold, and two alleles have approximately 8- to 12-fold increased risk of developing AD. While the E2 allele is associated with a decreased risk of developing AD, it increases the risk of cerebral amyloid angiopathy associated with hemorrhagic phenotype, the common small vessel disease in the elderly caused by beta-amyloid deposition at the leptomeningeal vessel [9].

While the *APOE* gene is a major genetic risk factor, several other genes have been implicated in genome-wide association studies (GWAS) such as clusterin (*CLU*), phosphatidylinositol binding clathrin assembly protein (*PICALM*), triggering receptor expressed on myeloid cells 2 (*TREM2*), complement receptor type 1 (*CR1*), protein kinase *D3* (*PRKD3*)*/NDUFAF7*, ATP binding cassette subfamily A member 7 (*ABCA7*), major histocompatibility complex, class II, DR beta 5 (*HLA-DRB5*)/DR beta 1 (*DRB1*), sortilin related receptor 1 (*SORLI1*), and numerous other loci have been identified from genome-wide study associated with LOAD [10,11,12,13,14]. These loci are involved in immunity, inflammation, regulating fat and protein metabolism, and transport both within the central nervous system and systemically. Co-pathology often exists in older adults, not only in extracellular amyloid, neuritic, and neurofibrillary plaque, but TAR DNA binding protein 43 (TDP-43) deposition, alpha-synuclein, and vascular pathology are also commonly seen.

### Frontotemporal dementia

Frontotemporal dementia (FTD) is characterized by degeneration of the frontal and/or temporal lobes, leading to impairments in social behavior, personality, aphasia, and, in some cases, features of motor neuron disease or Parkinsonism. In most cases, clinicopathological investigations have consistently identified tau or TDP-43 as primary pathologies. Approximately 20% of patients exhibit autosomal-dominant inheritance patterns and among patients with behavioral variant FTD, particularly those with concurrent motor neuron disease, inherited conditions are more prevalent [15, 16].

The most frequently observed genetic mutations associated with familial FTD involve microtubule-associated protein tau (*MAPT*), progranulin (*GRN*), and chromosome 9 open reading frame 72 (*C9orf72*) hexanucleotide repeat expansion [17]. Although less common, additional genes such as valosin containing protein (*VCP*), charged multivesicular body protein 2B (*CHMP2B*), and TANK-binding kinase 1 (*TBK1*) have been identified through molecular genetic studies [18].

### Parkinson's disease

Parkinson's disease (PD) is a neurodegenerative disorder characterized by the progressive loss of dopaminergic neurons in the substantia nigra pars compacta due to Alpha-synuclein misfolding, aggregation, and toxicity. It is manifested by rest tremor, rigidity, bradykinesia, and postural instability. While the exact cause of PD is not fully understood, genetic factors play a role, particularly in patients younger than 50 years old [19, 20].

A small number (10%) of PD is due to monogenic mutations. These mutations are associated with early-onset PD. Key genes associated with familial forms of PD are alpha-synuclein (*SNCA*), leucine-rich repeat kinase 2 (*LRRK2*), Parkinson's disease 2 (*PARK2*), *PARK7*, and PTEN-induced kinase 1 (*PINK1*) [19, 21]. Additionally, GWAS have identified multiple common genetic variants associated with an increased risk of developing PD. These variants were found near genes such as synuclein alpha (*SNCA*), microtubule-associated protein tau (*MAPT*), and glucosylceramidase (*GBA*) speculated to be regulatory toward the pathogenic genes [22]. However, genes identified through association studies require caution as these genes are also prevalent in the general population.

### Huntington's disease

Huntington's disease (HD) is characterized by basal ganglia degeneration, primarily affecting the caudate and putamen, with lesser involvement of the nucleus accumbens and globus pallidus [23]. This condition arises from CAG trinucleotide repeat expansion within the huntingtin gene. Alleles with over 26 repeats lead to the production of unstable proteins prone to further expansion across generations, while alleles with 36 repeats or more are associated with disease manifestation, characterized by protein aggregates in the cytoplasm and nucleus of the basal ganglia structures [24, 25]. HD typically manifests insidiously in adulthood, featuring movement abnormalities such as choreiform movements, psychiatric symptoms, cognitive impairment, and eventual dementia [26].

Other hereditary disorders exhibiting repeat expansions, such as Huntington disease-like 2 (HDL2) with CAG/CTG trinucleotide expansion in the junctophilin 3 (*JPH3*) gene, dentatorubral pallidoluysian atrophy (*DRPLA*) with CAG repeat expansions in atrophin-1 (*ATN1*) gene and mutation in *C9orf72* can present as phenocopies of HD [27,28,29].

### Autosomal dominant spinocerebellar ataxia

Autosomal dominant spinocerebellar ataxias (SCAs) represent a diverse group of adult-onset progressive neurodegenerative syndromes characterized by cerebellar dysfunction, presenting with symptoms such as ataxia, incoordination, nystagmus, and variable involvement of the brainstem, including oculomotor deficits, pyramidal signs, and occasionally extrapyramidal and cognitive impairments, depending upon the specific gene involved [30].

Currently, there are at least 49 subtypes of SCA, most of which result from a CAG trinucleotide repeat expansion and often exhibit familial aggregation. Among the commonly encountered subtypes are SCA1 (*ATXN1*), SCA2 (*ATXN2*), SCA3 (*ATXN3*), SCA6 (*CACNA1A*), SCA7 (*ATXN7*), SCA17 (*TBP*) and DRPLA (*ATN1*) [31]. SCA3, also known as Machado–Joseph disease, is the most prevalent subtype globally [31].

### Friedreich ataxia

Friedreich ataxia (FA) stands as the most prevalent form of hereditary ataxia, accounting for up to half of all cases. It follows an autosomal recessive inheritance pattern, typically manifesting in adolescence, with the age of onset inversely correlated to the number of GAA repeats [32]. This condition arises from mutations in the frataxin (*FXN*) gene, resulting in a loss of function and reduced expression of frataxin [33].

Frataxin plays a crucial role in maintaining the mitochondrial function, and its deficiency affects organs with high energy demands, such as the nervous system, heart, and pancreas [34]. Neurologically, FA patients present with limb ataxia due to dorsal column and cerebellar dysfunction, peripheral sensory axonal neuropathy, dysarthria, and progressive visual and auditory impairments [35, 36]. However, the clinical presentation can vary widely based on the number of repeats and the age of onset. Non-neurological manifestations may include hypertrophic cardiomyopathy and diabetes mellitus [32].

### Myotonic dystrophy

Myotonic dystrophy is the most prevalent form of inherited muscular dystrophy. It is characterized by muscle weakness and myotonia and involves non-neurological organs, such as cataracts and cardiac abnormalities, including cardiomyopathy and conduction disorders.

Two distinct types of myotonic dystrophy exist: type 1 and type 2 (DM1 and DM2). Both types result from the repeated expansion of CTG, with DM1 in the dystrophia myotonica protein kinase (*DMPK*) gene and DM2 in the zinc finger 9 protein (*ZNF9*) gene [37,38,39]. They diverge in age of onset, with DM1 typically presenting at a younger age and partly dependent on the number of CTG repeats. Clinically, weakness in DM1 predominantly affects the facial muscles, resulting in a characteristic long face and hollow cheeks, along with involvement of the sternocleidomastoid, forearm, intrinsic hand muscles, and ankle dorsiflexors [40, 41]. Conversely, DM2 primarily involves finger flexors, proximal muscles, and neck flexors [37].

### Duchenne and Becker muscular dystrophy

Duchenne muscular dystrophy (DMD) and Becker muscular dystrophy (BMD) are genetic muscle disorders causing muscle fiber degeneration caused by mutations in the dystrophin (*DMD*) gene. However, they differ in terms of severity depending on the mutation. These disorders are X-linked recessive genetic disorders, thus, they commonly affect young male patients [42].

#### DMD

Mutations in the dystrophin gene cause DMD, which are often large deletions, duplications, or frame-shift mutations that disrupt the gene's reading frame. As a result, no truncated, nonfunctional dystrophin protein is produced [43]. This is a severe mutation causing a loss of muscle fiber integrity, and symptoms usually appear in early childhood wherein the affected individuals experience progressive muscle weakness [20]. Most individuals with DMD are wheelchair-bound by their early teens, and the condition can lead to severe muscle weakness with pseudohypertrophy, dilated cardiomyopathy, conduction abnormalities, and severe scoliosis affecting the respiratory functions [44].

#### BMD

Mutations in the dystrophin gene also cause BMD, but the mutations in BMD are typically milder. They may include mis-sense mutations, in-frame deletions or duplications, or other mutations that allow for some production of partially functional dystrophin protein. Some functional dystrophin protein is produced, though it may be insufficient during adulthood [43].

### Spinal muscular atrophy

Spinal muscular atrophy (SMA) is a genetic neuromuscular disorder characterized by degeneration of the anterior horn cells in the spinal cord and motor nuclei in the lower brainstem, leading to symmetrical proximal muscle weakness and atrophy. SMA is an autosomal recessive inheritance disease primarily caused by mutations in the survival motor neuron 1 (*SMN1*) gene on chromosome 5 [45]. Deficiency in mRNA synthesis leads to the death of motor neurons. The severity of SMA is largely determined by the number of functional copies of the *SMN2* gene to compensate for *SMN1* mutation and correlates inversely with *SMN2* copies. The classification was done according to the age of presentation and ranked from 0 to 4 from prenatal to adult-onset [46]. Early diagnosis through genetic testing is crucial for the initiation of therapies.

### Amyotrophic lateral sclerosis

Amyotrophic lateral sclerosis (ALS) is a neurodegenerative disorder that primarily affects the motor neurons in the brain and spinal cord, resulting in muscle weakness and paralysis. Most ALS cases (approximately 90%–95%) are considered sporadic, and the exact causes are poorly understood; various genetic mutations and risk factors may contribute to its development. Approximately 5%–10% of ALS cases have a genetic link within the family, and several genes have been identified as responsible for familial ALS, including superoxide dismutase 1 (*SOD1*), *C9orf72*, fused in sarcoma (*FUS*), less commonly transactive response DNA binding protein (*TARDBP*), ubiquilin-2 (*UBQLN2*), *VCP*, and *TBK1* [47, 48]. Some individuals with familial ALS may have a family history of FTD, as these conditions share similar genetic mutations.

### Hereditary motor and sensory neuropathy

Hereditary motor and sensory neuropathy (HMSN) also known as Charcot–Marie–Tooth (CMT) disease, is a group of inherited neurological disorders that affect the peripheral nerves, leading to muscle weakness and sensory loss [49]. Over 100 genetic mutations cause CMT, which can be inherited in different ways, including autosomal dominant, such as mutation in peripheral myelin protein 22 (*PMP22*) and myelin protein zero (*MPZ*), autosomal recessive mitofusin 2 (*MFN2*), gang-lioside-induced differentiation-associated protein 1 (*GDAP1*), SH3 domain and tetratricopeptide repeats 2 (*SH3TC2*), and X-linked inheritance patterns, including gap junction beta 1 (*GJB1*) [50].

CMT can vary in severity and age of onset. It typically presents with common features such as distal muscle weakness, atrophy, and numbness, which often progress over time [51]. Genetic testing is important for definitive diagnosis, prognosis, and genetic counseling.

### CADASIL and CARASIL

Cerebral small vessel diseases are a group of disorders causing vascular abnormalities; two known hereditary vascular disorders causing infarctions and white matter changes are autosomal dominant arteriopathy with subcortical infarcts and leukoencephalopathy (CADASIL), and cerebral autosomal recessive arteriopathy with subcortical infarcts and leukoencephalopathy (CARASIL). However, genetic mutation and pathophysiological changes at the molecular level vary widely. The prevalence of CADASIL is 2–5/100,000, and 5,000 cases are reported for CARASIL [51].

#### CADASIL

CADASIL is an autosomal dominant genetic disorder characterized by accumulating granular osmophilic material (GOM) in the brain's small blood vessels. CADASIL is caused by mutations in the Notch3 homolog protein 3 (*NOTCH3*) gene on chromosome 19, which encodes a transmembrane receptor called Notch3, which is crucial for signaling in blood vessels. The mutations are typically point mutations, substituting a single nucleotide and producing an abnormal Notch3 protein [52]. The mutant Notch3 protein is thought to lead to the aggregation and accumulation of GOM in the walls of small blood vessels in the brain. This leads to progressive hypertrophy of the vessel's smooth muscle cells. Eventually, stenosis and dysfunction result in manifestations such as migraines with aura, mood disturbances (depression), vascular dementia, or small vessel infarctions between the ages of 35 and 55 [53]. The characteristic features are multiple lacunar infarctions, leukoencephalopathy, especially at the anterior temporal lobe, and multiple deep cerebral microbleeds (CMBs) with no traditional risk factor for atherosclerosis. The management of patients with CADASIL comprise intensive blood pressure control, avoiding smoking, and balancing the risks and benefits of antithrombotic agents [51].

#### CARASIL

CARASIL is inherited in an autosomal recessive manner. The underlying genetic cause of CARASIL is mutations in the high-temperature requirement A1 (*HTRA1*) gene located on chromosome 10 [54]. The *HTRA1* gene encodes the HtrA serine protease 1 protein, which regulates transforming growth factor beta (*TGFB*). With loss or reduced activity, TGFB goes unchecked in the smooth muscle cell layer and alters the integrity of the blood vessel walls [55]. The mutations can be missense or splicing in the *HTRA1* gene [54]. CARASIL is associated with a range of clinical symptoms, including recurrent subcortical infarcts and cognitive decline, but usually milder than CADASIL; other findings may include alopecia and skeletal abnormalities [55]. The common neuroimaging of CARASIL comprise confluent white matter changes in pons/middle cerebral peduncle, frontal white matter, anterior temporal lobe, external capsules and thalami; lacunar infarctions; deep CMBs; and cerebral atrophy [54].

### Mitochondrial disorders

Mitochondrial disorders are a group of genetic disorders causing mitochondrial dysfunction. Mitochondria are intra-cellular organelles, a powerhouse of the cell, found in almost all cells except red blood cells, and disorders can lead to a wide range of symptoms affecting various organs and tissues throughout the body. The genetics of mitochondrial disorders are unique because nuclear DNA (nDNA) and mitochondrial DNA (mtDNA) govern mitochondrial function, and both are present throughout life. Disorders of the brain can mimic other non-genetic disorders and include multisystem disorders, encephalopathy, myelopathy, myopathy, and presentation can be from acute to chronic or attack with spontaneous remission [56, 57].

#### mtDNA

mtDNA comprises 16,596 base pairs and is inherited exclusively from the mother, unlike nDNA, which is inherited from both parents. mtDNA encodes a subset of the proteins needed for mitochondrial function. Mutations in mtDNA can affect aerobic metabolism through metabolism and respiratory chain dysfunction, including complex I, III, IV, and V, or by disrupting mtDNA maintenance and leading to apoptosis. mtDNA has a high mutation rate due to a lack of histone, and sporadic germ cell mutations occur. However, due to heteroplasmy (mixture of pathogenic and normal mtDNA), penetrance and degree of severity vary even with the same pathogenic mutations [58]. They are responsible for 9 of 10 cases of mitochondrial disorders and the remaining due to nDNA mutation [59]. Diseases with mtDNA mutation include mitochondrial encephalopathy lactic acidosis stroke-like episodes (MELAS), myoclonic epilepsy with ragged red fibers (MERRF), Leber's hereditary optic neuropathy (LHON), chronic progressive external ophthalmoplegia (CPEO), and Kearns–Sayre [60]. They can also cause isolated myopathy, exercise intolerance, or respiratory muscle weakness.

#### nDNA

Most genes involved in mitochondrial protein are encoded by the nucleus, and not in mtDNA except for the polypeptide components of the respiratory chain. It was earlier believed that mitochondrial disorder caused by nDNA tended to cause disease in the pediatric population, but it is now clear that both mtDNA and nDNA can cause disease throughout life [58]. Diseases with nDNA mutation include myopathy or encephalomyopathy related to coenzyme Q10 deficiency, mitochondrial neurogastrointestinal encephalomyopathy (MNGIE), Leigh syndrome, and a whole host of syndromes associated with mutations such as polymerase gamma (POLG) enzyme, complex 1–5 and carnitine palmitoyltransferase (CPT) [60].

### Current and future therapy in neurogenetics

Gene therapy is a ground-breaking approach to neurology and neurogenetics. Neurologists need to understand that many genes affect the brain since the developmental phase, and early diagnosis will be imperative once treatments are available. Current gene therapy is based on introducing, modifying, or silencing specific genes within an individual's cells to reverse the disruption of the cell's processes.

The fundamentals of gene therapy involve identifying the precise genes responsible for the disorder; this is followed by the delivery of therapeutic genes into target cells, which require vectors, such as adeno-associated viruses (AAVs) and lentiviruses, with an ability to penetrate the blood–brain barrier and transduce neural cells. Another approach is to regulate existing genes, often involving promoters and regulatory elements.

### Current disease-modifying therapy

#### AD

AD is influenced by multiple genes and environmental factors, making it challenging for targeted treatment. The pathogenesis of AD remains a subject of ongoing debate, with the amyloid beta hypothesis being the most widely accepted theory as the initiating or potentiating of the disease [61,62,63].

Despite extensive research, no curative treatment is currently available for AD. However, disease-modifying treatments that target beta-amyloid protein, such as lecanemab and donanemab, have shown promising results in slowing cognitive decline [64, 65]. These treatments utilize a monoclonal antibody approach to remove the pathogenic protein.

#### SMA

SMA is caused by mutations in the SMN1 gene. Gene therapy with Onasemnogene abeparvovec delivers functional DNA of SMN1 using AAV [66]. Nusinersen increases the production of the *SMN2* gene by modifying splicing using an antisense oligonucleotide [67]. Finally, Risdiplam modifies SMN2 premRNA and increases the SMN protein production [68].

#### Duchenne and BMD

Both are characterized by mutations in the *DMD* gene. Techniques such as exon skipping with Eteplirsen in patients with deletion at a location feasible for exon 51 skipping may improve function with others in its class such as Golodirsen and Viltolarsen; antisense oligonucleotide targeting exon 53 [64,65,66]. Ataluren promotes the readthrough of nonsense mutation which benefits patients with stop mutation [67]. Finally, Delandistrogene moxeparvovec is an AAV-containing dystrophin protein [67].

#### ALS

Disorder of protein and energy regulation such as altered RNA processing, excitotoxicity, and mitochondrial dysfunctions surrounding motor neuron degeneration is found in ALS [69, 70]. Altered RNA processing has emerged as a focal point in understanding ALS pathogenesis, with mutations leading to mislocalization of TDP-43 and FUS, consequently impairing RNA quality control. Pathologic variants in SOD1 in familial ALS also contribute to toxic protein misfolding [71].

Recent therapeutic advances have provided targeted treatments for specific genetic subtypes of ALS. Tofersen, an antisense oligonucleotide administered intrathecally, showed a slower decline in the ALS functional rating scale, vital capacity, and grip strength. It has been approved for ALS associated with SOD1 pathogenic mutations [72]. For other ALS patients, treatment options are limited. Riluzole is commonly prescribed; although its exact mechanism of action remains unclear, it is thought to mitigate glutamate excitotoxicity [73].

There are additional pharmacological interventions for another ALS pathogenesis. Edaravone, a free radical scavenger, aims to reduce oxidative stress, while sodium phenylbutyrate-taurursodiol modulates adaptive stress responses in the endoplasmic reticulum and maintains mitochondrial integrity, thereby reducing neuronal apoptosis [74, 75]. These treatments are combined with symptomatic and palliative care.

Gene therapy offers unprecedented opportunities to treat genetic disorders and other diseases with a genetic component. Other than the mentioned techniques, clustered regularly interspaced short palindromic repeats (CRISPR)/CRISPR-associated protein 9 (Cas9) or CRISPR-Cas9, is a bacterial defense mechanism against plasmid transfer with an ability for the CRISPR region to generate short RNA strands by transcription and Cas9 to disrupt and insert RNA, thereby correcting mutation or disrupting disease genes precisely [76, 77]. Additionally, emerging techniques from Cas9, like base editing and prime editing, offer even greater precision in gene modification [76, 77]. However, challenges related to off-target that lead to unknown mutation in random areas are still a concern and necessitate ongoing research to optimize these techniques and ensure their long-term safety. Nevertheless, gene therapy represents a paradigm shift in treating neurogenetic disorders and the transformative potential that gene therapy holds for the future of neurogenetics.

## Neurogenetic clinics in resource-limited settings

Genetic testing has several roles for neurologists, such as diagnostic testing to confirm or rule out a suspected genetic disorder. Predictive testing is for those with a family member with a genetic disorder to determine a person's risk of developing the specific disorder. Pharmacogenomic testing tells you how the patient will react to certain medications, including efficacy, adverse drug reaction, and monitoring. Reproductive testing is related to genetic variants parents may carry to make decisions before, during, and after pregnancy.

Neurogenetics focus mainly on diagnostic and predictive testing, but significant challenges such as accessibility in resource-limited settings remain an issue. Despite these challenges, establishing neurogenetic clinics is crucial for early diagnosis, reduces resource use in various non-diagnostic investigations, and can provide opportunities for targeted therapies and better management of neurological disorders. These clinics can offer genetic counseling, educating patients and families about the condition, its inheritance patterns, and available support systems. Finally, the service will contribute valuable data to global genetic research and help us understand these conditions. The uniqueness of genetic variation in each ethnicity and region is crucial to complete the complexity of human genetics.

The issue of accessibility to genetic testing and technologies means that collaboration with regional and international laboratories is of utmost importance with the help of international funding. A lack of interest in genetics can impede the functioning of neurogenetic clinics, and training can address this issue, either online or in person. Although gene therapy is relatively expensive, it is still crucial for patients and relatives who are suffering from genetic diseases. Some gene therapies could change the whole lives and fates of patients. Regional and international collaboration would fill the gap in this issue. This would lead to further research and advancement of technology in the field.

## Ethical considerations

The complex nature of genetic testing, its interpretation, and heritability raise important ethical issues for the clinician. With no exception, informed consent must be obtained before such testing, given the potential implications for the patient and their family. Genetic information can provide depth of information into the disease's short- and long-term consequences, raising important considerations regarding patient autonomy and privacy, particularly given the hereditary nature. Neurologists may find it necessary to become genetic counsellors themselves before carrying out these tests and emphasize that genetic testing is ultimately for their family as well.

Nevertheless, the conflict between respecting patient autonomy and privacy could still occur, and there may be instances where withholding information from at-risk family members could deprive them of essential healthcare information and the opportunity to make informed decisions regarding their health and family planning. There is no definitive answer for each scenario, but one must try to place ethical consideration into these situations, involve other specialties, and involve patients and their families to try to reach the best possible solution, weighing all the factors, including the severity of the mutation. This solution must always be guided by the best interests of the patient and their families and supported by evidence and ethical standards. It is essential to acknowledge that individuals have the right not to be informed about genetic testing results, and this preference should be respected while ensuring that discussions are made with compassion and respect for the wishes and well-being of all involved parties.

## Conclusion

Genetics has changed the landscape of neurology from neuro-development and neuronal function to neurological disorders. Genetic research has deepened our understanding and provided new avenues for diagnosis and treatment, enabling personalized medicine approaches. Understanding the rapidly evolving field of genetics and neurology will ultimately improve care for the patient.
